# Genome-wide identification of the HD-ZIP IV gene family in *Pogostemon cablin* and its association with glandular hair development

**DOI:** 10.3389/fpls.2025.1580351

**Published:** 2025-05-12

**Authors:** Shuqi Xie, Yiling Li, Huiting Liao, Wenyi Chen, Yuqi Zhang, Xiaoming Yan, Hongyi Zhang, Mengling He, Hanjing Yan

**Affiliations:** ^1^ College of Traditional Chinese Medicine, Guangdong Pharmaceutical University, Guangzhou, Guangdong, China; ^2^ Office of the Drug Testing Institute, Guangdong Institute for Drug Control, Guangzhou, Guangdong, China

**Keywords:** homeodomain leucine zipper IV, genome-wide analysis, *Pogostemon cablin*, plant hormones, glandular trichomes

## Abstract

**Background:**

HD-ZIP IV transcription factors are a plant-specific subgroup of the homeodomain-leucine zipper (HD-ZIP) family, known to regulate epidermal cell differentiation. While their functions have been studied in many plant species, their roles in *Pogostemon cablin* (patchouli) remain largely unexplored.

**Methods:**

We conducted a comprehensive genome-wide identification and analysis of *HD-ZIP IV* genes in patchouli. Phylogenetic relationships, gene structures, conserved motifs, and promoter cis-elements were examined. Expression patterns under hormone treatments were analyzed using qRT-PCR. Correlation analyses and virus-induced gene silencing (VIGS) were employed to assess gene function in glandular trichome development.

**Results:**

A total of 38 *HD-ZIP IV* genes (*PcHDZIV1–PcHDZIV38*) were identified and classified into six subfamilies. Gene structure and motif analyses revealed conserved features within subgroups. Promoter analysis indicated widespread involvement in light, hormone, and stress responses. Many *PcHDZIV* genes showed dynamic responses to exogenous hormone treatments (MeJA, IAA, SA). Notably, *PcHDZIV5* expression correlated strongly with glandular trichome density, and VIGS experiments confirmed its role in promoting trichome development.

**Discussion:**

Our findings suggest that hormone signaling may regulate *PcHDZIV5* expression, indirectly influencing glandular trichome formation in patchouli. This study lays a foundation for further functional characterization of *HD-ZIP IV* genes in patchouli and advances understanding of the molecular mechanisms underlying trichome development.

## 1 Introduction

*Pogostemon cablin* (Blanco) Benth., known in common parlance as patchouli, is a plant species from the Lamiaceae family, native to Southeast Asia (Murugan and Livingstone, 2010). Due to its unique fragrance and significant fixative properties, patchouli has become one of the main raw materials in the food and fragrance industries (Swamy and Sinniah, 2016). Additionally, patchouli is known to harbor several pharmacologically active secondary metabolites, including patchouli alcohol, patchouli ketone, β-patchoulene, and epoxy-patchoulene (Junren et al., 2021). However, as a field crop, patchouli is exposed to numerous biotic and abiotic stresses—such as insect pests, high light intensity, and drought—that notably affect its yield and overall quality. Glandular trichomes(GTs), as natural physical barriers in plants, defend against herbivores and insect pests by secreting toxic substances, thus mitigating biotic stresses (Kang et al., 2010; Rakha et al., 2017), and also reduce water loss by forming a waxy layer, protecting against abiotic stresses like drought and high salinity (Hegebarth et al., 2016; Busta et al., 2017). Moreover, GTs in patchouli are crucial for the synthesis of secondary metabolites, particularly in the production and storage of sesquiterpenes, such as patchouli alcohol, which directly influence the quality of the plant (Guo et al., 2013). Therefore, understanding the molecular processes governing GT development in patchouli is not only of great theoretical importance but also provides practical guidance for improving the quality and stress resistance of patchouli.

Belonging exclusively to higher plants, the HD-ZIP protein family constitutes a unique class of transcription factors. In higher plants, these proteins are essential for growth and development, adaptation to abiotic stresses, and the synthesis of specific secondary metabolites (Ariel et al., 2007). A common structural feature of HD-ZIP proteins is the presence of a homeodomain (HD) and a leucine zipper (LZ) domain that is tightly associated with it (Bürglin and Affolter, 2016). The HD-ZIP gene family is divided into four subfamilies: HD-ZIP I, HD-ZIP II, HD-ZIP III, and HD-ZIP IV, according to their DNA-binding specificity, gene architecture, conserved motifs, and functional characteristics (Elhiti and Stasolla, 2009).

HD-ZIP IV transcription factors, in addition to the HD and LZ domains, also possess a START domain and a SAD domain (Chew et al., 2013). Moreover, HD-ZIP IV proteins have the ability to specifically bind to cis-elements characterized by the core sequence CATT(A/T)AATG (Tron et al., 2001). HD-ZIP IV proteins typically function in the epidermal and sub-epidermal cells of plants, playing key roles in epidermal cell development and maintenance, anthocyanin accumulation and regulation, lipid transport, and drought stress responses (Chew et al., 2013). Comprehensive genome-wide analyses have revealed the presence of *HD-ZIP IV* genes in several plant species, such as *Arabidopsis thaliana* (Nakamura et al., 2006), tobacco (Zhang et al., 2019), tomato (Gao et al., 2015), maize (Javelle et al., 2011), cucumber (Fu et al., 2013), watermelon (Yan et al., 2022), and *Salvia miltiorrhiza* (Wan et al., 2024). *Arabidopsis* features 16 members within its HD-ZIP IV transcription factor family. Among them, *AtGL2* is expressed in leaf epidermal cells that will develop into GTs and in non-root hair cells of the root, playing a role in the initiation of GTs and the differentiation of epidermal cells in roots and seed coats. *ATML2* and *PDF2* are predominantly expressed in the outermost cells of the *Arabidopsis* ovule epidermis and apical meristem, where they play a crucial role in the regulation of epidermal cell development (Nakamura et al., 2006; Takada and Jürgens, 2007). In tobacco, 32 HD-ZIP IV family members have been identified. *HD-ZIP IV* genes were found to exhibit high expression levels in the epidermal layers of leaves, stems, and roots, as shown by tissue expression analysis, and are involved in epidermal development and adaptation to abiotic stresses (Zhang et al., 2019). The tobacco *HD-ZIP IV* gene *NtHDG2* promotes the synthesis of tobacco flavonoid alcohols (Wang et al., 2020). The gene knockout of the HD-ZIP IV transcription factor *wo* in tomato leads to defects in GT formation (Hua et al., 2021). Additionally, *SlHDZIV8* regulates Hairless-2 expression by interacting with the L1-box region of its promoter, thereby participating in the cytoskeletal organization and morphological development of GTs (Xie et al., 2020). In peppermint, *McHD-Zip3* forms a complex with the transcription factor McMIXTA to co-regulate the development and differentiation of GTs (Qi et al., 2022). In *Artemisia annua*, *AaHD8* interacts with *AaMIXTA1* to form an HD-ZIP IV/MIXTA complex, which in turn regulates the expression of *AaHD1*. *AaHD1* promotes GT initiation, thereby accelerating GT development in *A. annua* (Yan et al., 2017, 2018). In summary, members of the plant HD-ZIP IV gene family participate in various abiotic stress responses and epidermal differentiation processes through different regulatory mechanisms. Therefore, it is valuable to study their functional attributes in different plants.

However, due to the complex genetic background of patchouli, traditional breeding methods still face significant challenges in altering its traits. Gene-engineering-based improvement strategies have brought new hope for addressing this issue, but the prerequisite is the identification of key GT regulatory genes in patchouli. Given the important roles of *HD-ZIP IV* genes in plant growth, GT development, and stress responses, a comprehensive identification and functional analysis of the HD-ZIP IV gene family in patchouli is crucial for revealing their potential role in GT development and provides a foundation for subsequent research.

In this study, the *PcHDZIVs* was identified and analyzed at the genome-wide level, with a focus on their physicochemical traits, gene architecture, evolutionary relationships, synteny analysis, and cis-acting elements. Gene expression was also analyzed across various leaf positions and hormone treatments, and the relationship between gene expression and GT density was explored. Further, we validated the regulatory role of *PcHDZIV5* in GT development. In summary, this study enhances the understanding of GT development in patchouli and serves as a basis for advancing functional research on *PcHDZIVs* in this species.

## 2 Materials and methods

### 2.1 Plant materials and hormone treatments

The patchouli seedlings, grown in the field in Yingli Town, Leizhou City, Zhanjiang, Guangdong Province (N20°57′28″, E110°09′02″), were transplanted into plastic pots and transferred to Guangzhou, Guangdong Pharmaceutical University (N23°05′66″, E113°41′26″). Distilled water was used as the control group, while the leaves of patchouli were sprayed with 300 µM MEJA (Wang et al., 2019), 150 µM IAA (Yuan et al., 2021), and450 µM SA (Es-sbihi et al., 2020) until water droplets began to fall. The plants were then immediately covered with transparent plastic film (Wang et al., 2019; Li et al., 2024). The plastic film was removed 1.5 hours later. At 0, 3, 6, 12, 24, 48, and 72 hours post-treatment, the second pair of leaves were collected. Simultaneously, the first to fifth pairs of leaves, counted from the top of the plant, were collected from normally growing plants. To prepare for total RNA extraction, all samples were enclosed in aluminum foil, flash-frozen in liquid nitrogen, and stored at -80°C. Each sample consisted of three biological replicates.

### 2.2 RNA preparation and quantitative real-time PCR

The RNAprep Pure Plant Kit (DP432; TIANGEN Biotech) was used to extract total RNA from *Pogostemon cablin* leaves, following the guidelines provided by the manufacturer. cDNA was then synthesized by reverse transcription with the PrimeScript™ RT reagent Kit (RR047A, TAKARA). The qRT-PCR analysis was performed TB Green^®^ Premix Ex Taq™ II (RR820A; TaKaRa) on a QuantStudio™ 1 Plus Real-Time PCR instrument, and each sample was analyzed in triplicate. Relative expression levels were normalized using 18S rRNA as the internal reference. The 2^−ΔΔCT^ method was applied to analyze the relative expression data, with the expression of the control sample (0h) and the first pair of leaves normalized to 1 (Livak and Schmittgen, 2001).

### 2.3 Identification of *PcHDZIVs* in patchouli

To identify the *PcHDZIVs*, protein and nucleotide sequences of patchouli were downloaded from the National Genomics Data Center(NGDC)(https://ngdc.cncb.ac.cn/) as a reference database (Shen et al., 2022). The 16 HD-ZIP IV protein sequences from *Arabidopsis* obtained via the TAIR database served as templates. A local BlastP search (E < 1 × 10^-^¹^0^) was executed, followed by manual removal of redundant sequences, with only the longest sequence retained from each splicing variant. Candidate sequences were further analyzed using InterProScan to ensure the presence of HD, LZ, and START domains, while excluding the MEKHLA domain.

### 2.4 Gene structure and protein sequence analysis

The gene structure map and localization information of *PcHDZIVs* was predicted and constructed using TBtools software (V 2.119) (Chen et al., 2020). Use the MEME program (V 5.57) (http://meme-suite.org/tools/meme) to identify conserved motifs in the PcHDZIVs protein sequences and perform functional annotation using the Pfam database. The maximum number of motifs is set to 15, with other parameters set to default values. Set the maximum motif number to 15, with other parameters set to default values. The physical and chemical properties of the PcHDZIVs proteins were measured using the ProtParam tool on the ExPasy website (http://web.expasy.org/protparam/). The subcellular localization of PcHDZIVs proteins was predicted using the WoLF PSORT website (https://wolfpsort.hgc.jp/).

### 2.5 Promoter Cis-element analysis

Extract the 2000 base pairs of promoter sequence located upstream of the transcriptional start site of *PcHDZIVs*. Use the PlantCARE database (http://bioinformatics.psb.ugent.be/webtools/plantcare/html/) to predict the cis-acting elements of the promoter sequence (Lescot et al., 2002).

Additionally, previous studies have reported the presence of L1-Box elements in the promoters of *HD-ZIP IV* genes in *Arabidopsis thaliana* (Roodbarkelari and Groot, 2017) and *Artemisia annua* (Yan et al., 2018). These elements not only serve as binding sites for HD-ZIP IV transcription factors but may also act as regulatory targets for other members of the HD-ZIP IV family. Based on this, the present study further screened for L1-Box elements in the promoter sequences of *PcHDZIVs* genes. All analysis results were visualized using TBtools software (V2.119).

### 2.6 Phylogenetic tree and collinearity analysis

To further investigate the phylogenetic relationships among HD-ZIP IV family members from different plant species, a phylogenetic tree was constructed using partial HD-ZIP IV protein sequences from *Arabidopsis* (Nakamura et al., 2006), Tomato (Gao et al., 2015), Watermelon (Yan et al., 2022), *Salvia miltiorrhiza* (Wan et al., 2024), *Oryza sativa* (Ito et al., 2003),*Artemisia annua* (Yan et al., 2017, 2018), and patchouli. The phylogenetic tree was generated using MEGA 10 software (Neighbor-Joining, Jones-Taylor-Thornton model, 1000 bootstrap replicates) and visualized and enhanced using the Evolview online tool (http://www.evolgenius.info/evolview/). To study the selective pressure acting on each pair of *PcHDZIVs*, synonymous (Ks), non-synonymous (Ka) substitutions, and the Ka/Ks ratio were calculated using KaKs_Calculator 2.0 (https://sourceforge.net/projects/kakscalculator2/). Collinearity analysis of *HD-ZIP IV* genes within the patchouli genome, as well as between Arabidopsis and Salvia miltiorrhiza, was performed using the MCScanX software. Collinearity maps within and between genomes were visualized using Circos software (V3.91) and TBtools software (V2.119).

### 2.7 Expression pattern of HD-ZIP IV genes in different tissues of patchouli

To investigate the expression profile of *PcHDZIVs*, transcriptome datasets of patchouli were downloaded from the NCBI database (PRJNA606892, PRJNA607886). RNA-seq reads were quantified using the RSEM (RNA-Seq by Expectation-Maximization) software (Li and Dewey, 2011), and heatmaps were generated using TBtools software.

### 2.8 Virus-induced gene silencing assay

A 300 bp coding sequence (CDS) fragment of *PcHDZIV5* was cloned into the pTRV2 vector to generate pTRV2-*PcHDZIV5*. The Agrobacterium tumefaciens strain GV3101 carrying the target construct was then infiltrated into the abaxial side of the second pair of patchouli leaves (Li et al., 2024). After 14 days, the second pair of leaves was collected for RT-qPCR analysis to assess the expression level of the target gene, and the number of GTs was quantified.

### 2.9 Glandular trichome count and pearson correlation analysis

The collected patchouli leaf samples from different leaf positions were preserved in FAA fixation solution. A 1 cm² leaf tissue was excised, treated with chloral hydrate for dehydration and clearing, and then mounted on a temporary slide (Heinrich et al., 2002). GTs density was observed under an optical microscope at 100× magnification, with GTs counted within three randomly selected fields of view per slide. Each sample was analyzed with three biological replicates. The data were analyzed and visualized with Origin 2022 software, based on the qRT-PCR results and GT count data. The Pearson correlation coefficient (PCC) and p-value between the expression levels of *PcHDZIVs* and GT numbers in different leaf positions were calculated.

Leaves subjected to VIGS treatment were stained with 0.2% Rhodamine B solution for 30 min, followed by three washes with distilled water. Temporary slides were prepared and observed under a fluorescence microscope (OLYMPUS, IX73) at 40× magnification. GTs were counted within five randomly selected fields of view per slide, with each sample analyzed in three biological replicates (Wang et al., 2022).

## 3 Results

### 3.1 Identification and physicochemical properties of patchouli HD-ZIP IV genes

Using 16 *Arabidopsis* HD-ZIP IV protein sequences as templates, a BLAST search was conducted in the patchouli genome database. After filtering out redundant sequences and MEKHLA domain-containing genes, 38 *PcHDZIVs* were successfully identified in the patchouli genome (E < 1 × 10^-^¹^0^). These genes were further analyzed using InterProScan to ensure the presence of the HD, LZ, and START domains, while confirming the absence of the HD-ZIP III-specific MEKHLA domain. Based on their chromosomal positions, these patchouli *HD-ZIP IV* genes were named *PcHDZIV1* to *PcHDZIV38*. In this study, the identified PcHDZIVs proteins had an amino acid length ranging from 615 to 817, molecular weight (MW) from 69.35 kDa to 91.1 kDa, and isoelectric point (pI) ranging from 5.23 to 8.88. PcHDZIV6 and PcHDZIV34 were identified as the largest and smallest members of the subfamily, respectively. Various characteristics of the 38 identified PcHDZIVs protein, including predicted amino acid count, isoelectric point, molecular weight, fat index, instability index, hydrophobicity average, and subcellular localization prediction, are summarized in **Supplementary Table 1**.

### 3.2 Gene structure and motif analysis of patchouli *HD-ZIP IV* genes

**Figure 1:**
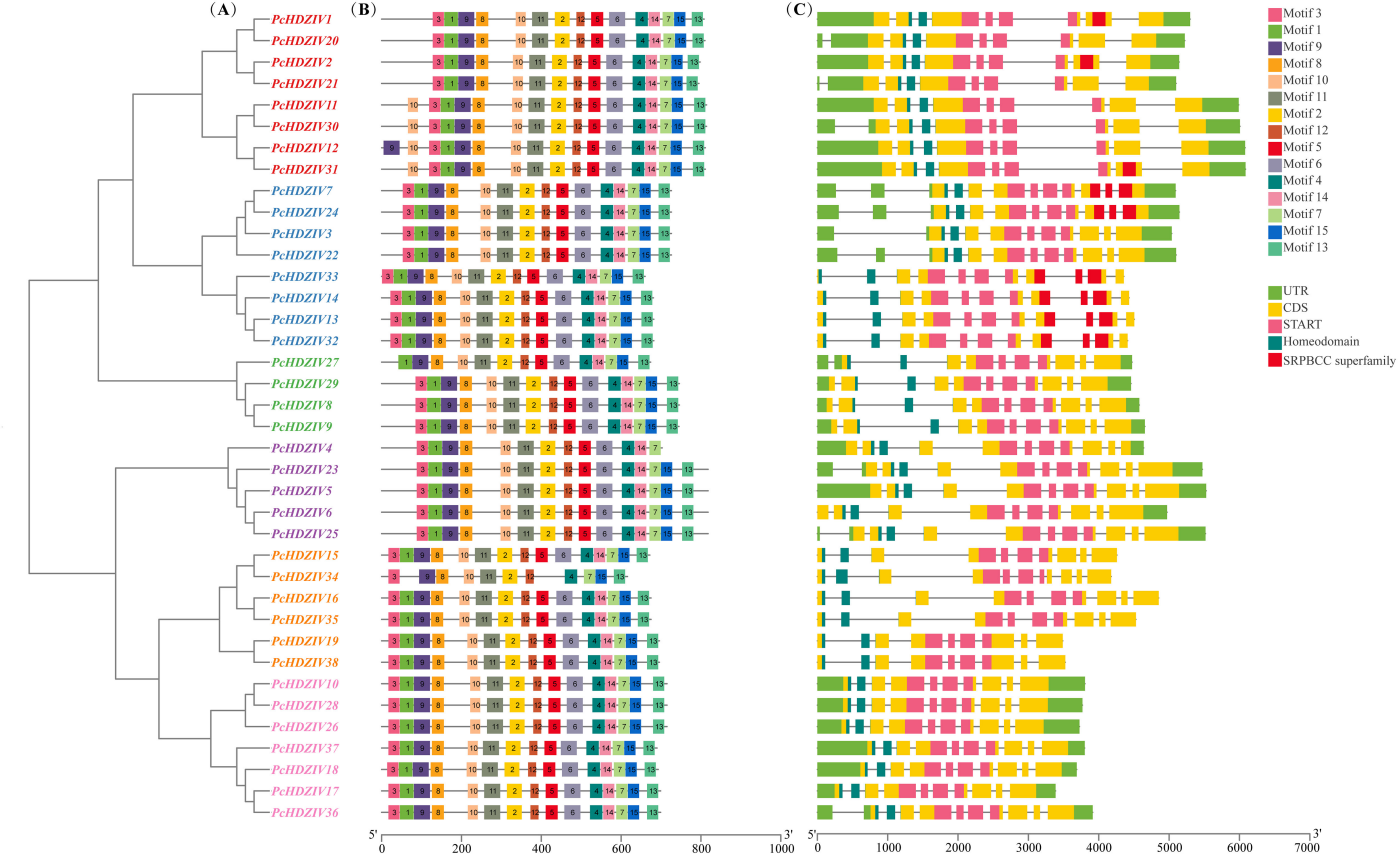
Gene structure and conserved motif analysis of *HD-ZIP IV* genes in patchouli. **(A)** Unrooted phylogenetic tree of *HD-ZIP IV* genes in Patchouli. Based on phylogenetic relationships, the genes can be divided into six clades, with genes of the same color representing one subfamily. **(B)** Conserved motif analysis of *HD-ZIP IV* genes in Patchouli. Boxes of different colors represent distinct conserved motifs. **(C)** Gene structure of *HD-ZIP IV* genes in Patchouli. Light green regions indicate UTRs, yellow regions represent CDSs, black horizontal lines denote introns, pink regions indicate START domains, dark green regions represent HD domains, and red regions indicate SRPBCC superfamily domains.

A phylogenetic tree comprising the 38 PcHDZIVs protein sequences was generated using the Neighbor-Joining (NJ) approach. The results indicate that these proteins can be divided into six branches: PcHDZIV 1, 2, 11, 12, 20, 21, 30, 31; PcHDZIV 3, 7, 13, 14, 22, 24, 32, 33; PcHDZIV 8, 9, 27, 29; PcHDZIV 4, 5, 6, 23, 25; PcHDZIV 15, 16, 18, 19, 34, 38; and PcHDZIV 10, 17, 18, 26, 28, 36, 37 (**Figure 1A**). Fifteen motifs were predicted for the PcHDZIVs proteins using MEME software. Motifs 1, 3, and 9 correspond to the HD domain, while motifs 2, 4, 5, 6, 10, 11, and 12 correspond to the START domain. All 38 PcHDZIVs proteins contain the characteristic domains typical of the HD-ZIP IV family. While motif compositions are generally uniform within the same subfamily, some proteins display differences. For example, PcHDZIV 27 lacks motif 3, PcHDZIV 4 lacks motif 13, and PcHDZIV 34 lacks motifs 1 and 5 (**Figure 1B**).

To further elucidate the role of *PcHDZIVs* during development, we conducted a detailed analysis of their exon-intron structures. The analysis indicates that the exon count within this gene family varies between 9 and 12. Among the 38 genes analyzed, 7 contain 9 exons, 12 have 10 exons, 14 have 11 exons, and 5 contain 12 exons (**Figure 1C**). These findings indicate that genes within the same subfamily exhibit strikingly similar structural traits, a pattern that is consistent with the phylogenetic analysis results.

### 3.3 Promoter Cis-elements analysis of patchouli *HD-ZIP IV* genes

**Figure 2:**
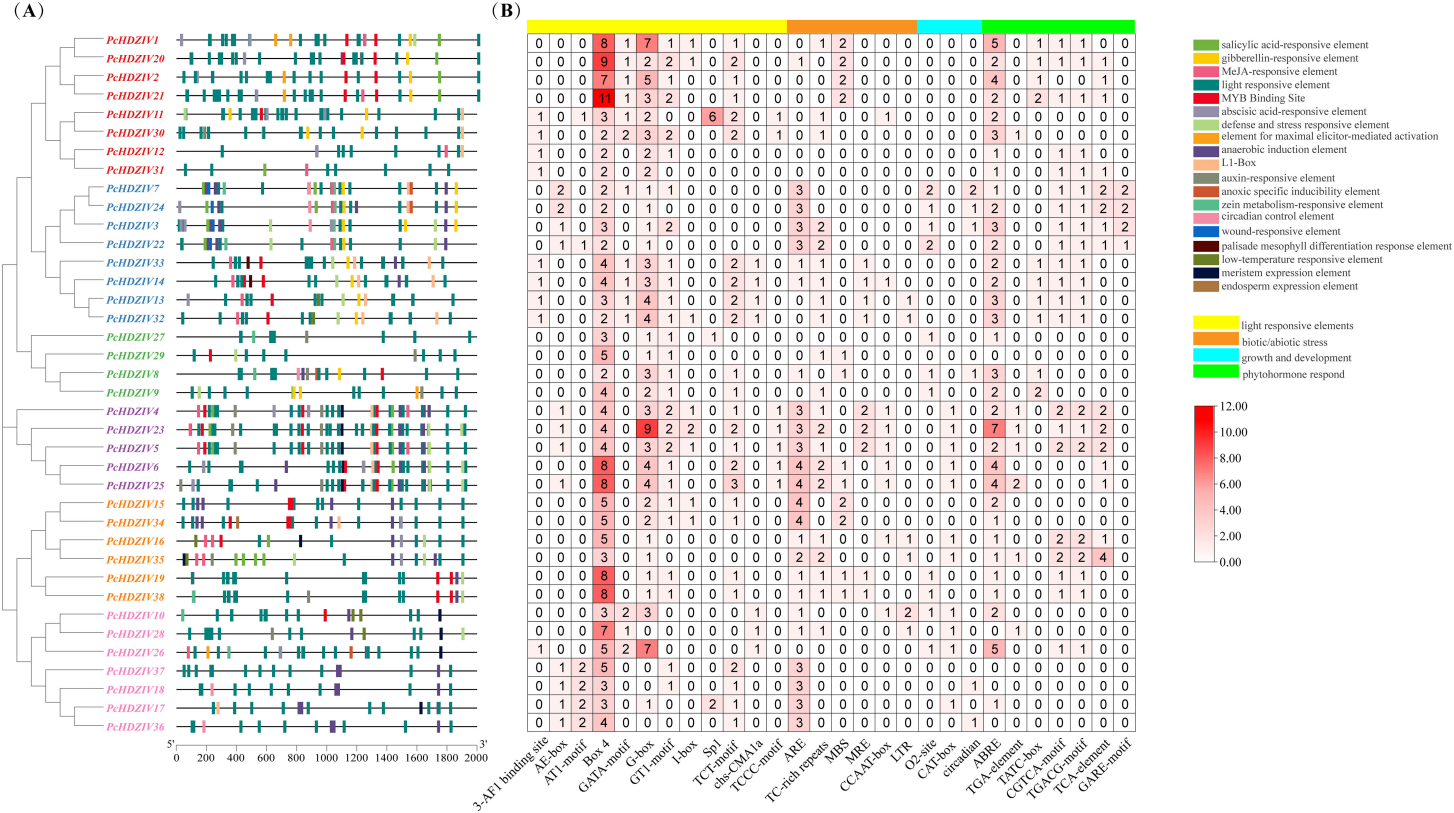
Analysis of cis-acting elements in the promoters of *HD-ZIP IV* genes in patchouli. **(A)** Location information of cis-acting elements in the promoters of *HD-ZIP IV* genes in Patchouli. Different cis-acting elements are represented by boxes of different colors. **(B)** Classification of cis-acting elements in the promoters of *HD-ZIP IV* genes in Patchouli. The gradient colors and corresponding numbers in the grid indicate the quantity of cis-acting elements in the gene promoters.

To further explore the functions and regulatory mechanisms of *PcHDZIVs*, we studied the cis-regulatory elements(CREs) in the promoters of *PcHDZIVs*. Twenty-eight types of CREs involving eighteen functions were identified (**Figure 2**). The elements related to light response were the most abundant, comprising twelve types. Hormone-related elements included seven types, those involved in stress response numbered six, and those related to growth and development were three (**Figure 2B**). Light-responsive cis-elements were identified in all *PcHDZIVs*, with Box 4 being universally present and frequently observed in multiple copies. In terms of hormone response, thirty-three genes contained abscisic acid (ABA), twenty-one genes contained methyl jasmonate (MeJA), fifteen genes contained salicylic acid (SA), and twelve genes contained auxin (IAA) related cis-acting elements. This indicates that *PcHDZIVs* are extensively involved in hormone response processes. It is noteworthy that all genes, with the exception of *PcHDZIV12*, *PcHDZIV27*, *PcHDZIV28*, and *PcHDZIV31*, contained cis-elements associated with stress responses. The presence of these hormone and stress-related elements in the *PcHDZIVs* promoters suggests that the transcriptional profiles of the *PcHDZIVs* might vary with hormonal and abiotic stress changes.

Previous studies have shown that HD-ZIP IV family members are not only regulated by plant hormones but can also influence the expression of other members through intra-family autoregulatory mechanisms. For example, in *Artemisia annua*, AaHD8 directly binds to and activates the AaHD1 promoter, thereby participating in GT development (Yan et al., 2018). Based on this, the present study screened the promoter regions of the HD-ZIP IV gene family in Patchouli for L1-Box elements. The results revealed that L1-Box binding sites were present in the promoters of 16 *PcHDZIV* genes. Notably, *PcHDZIV14*, *PcHDZIV32*, and *PcHDZIV33* each contained two L1-Box elements in their promoters. These findings suggest the potential existence of an intra-family regulatory mechanism among *HD-ZIP IV* genes in Patchouli.

### 3.4 Phylogenetic analysis of patchouli *HD-ZIP IV* proteins

**Figure 3:**
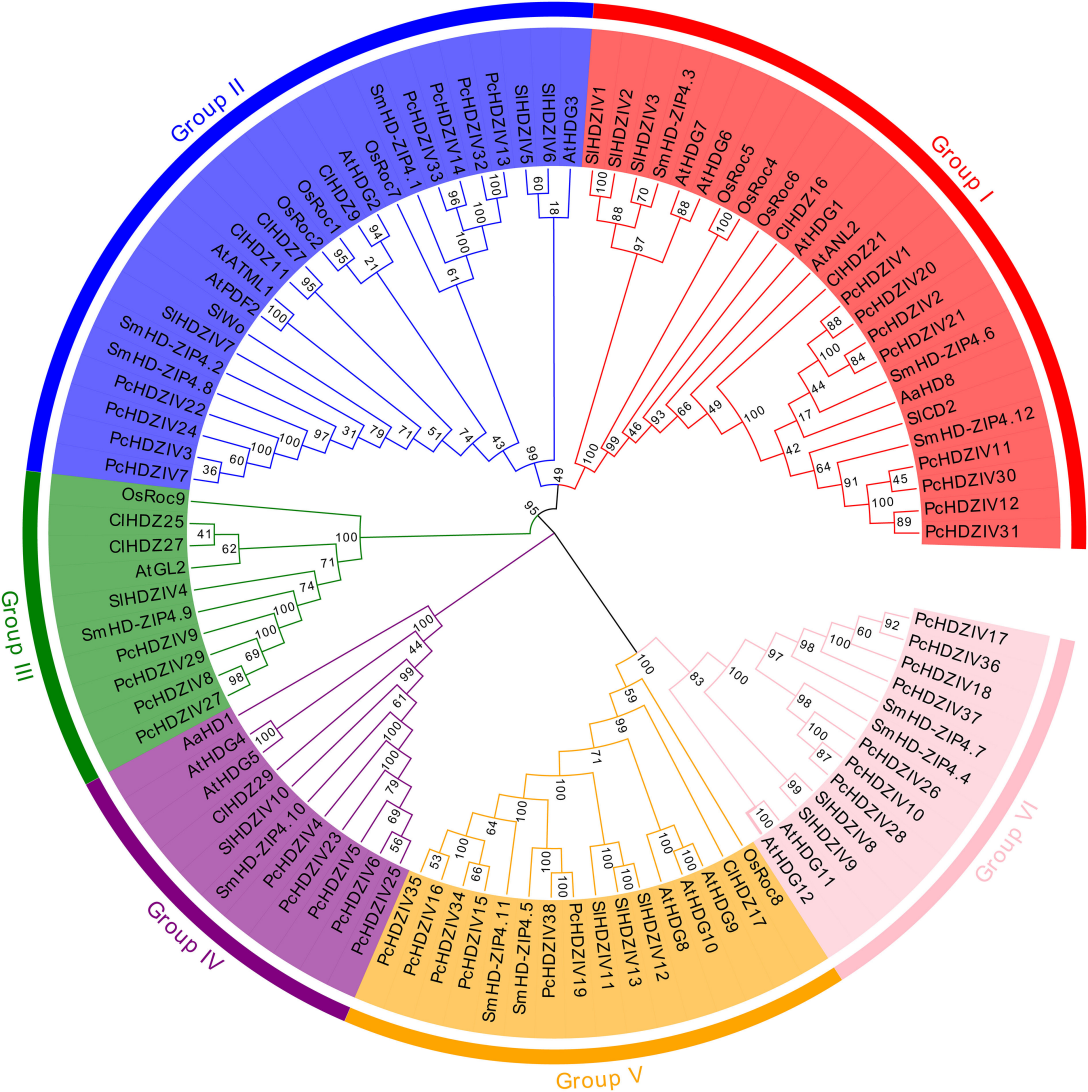
Phylogenetic tree constructed from HD-ZIP IV proteins in patchouli., *Arabidopsis thaliana* (At), *Solanum lycopersicum* (Sl), *Citrullus lanatus* (Cl), *Artemisia annua* (Aa), *Oryza sativa* (Os), and *Salvia miltiorrhiza* (Sm). The phylogenetic tree was constructed using the neighbor-joining method in MEGA 10 software (1000 bootstrap replicates). Different colors are used to distinguish various subclades, labeled as Group I to VI.

To further explore the evolutionary ties between PcHDZIVs proteins and HD-ZIP IV proteins from other species, we constructed a phylogenetic tree including 38 patchouli PcHDZIVs proteins and 62 HD-ZIP IV protein sequences from species such as *Arabidopsis*, tomato, watermelon, *Artemisia annua*, *Salvia miltiorrhiza*, and rice. These proteins, as revealed by the results, are grouped into six evolutionary clusters, referred to as I to VI (**Figure 3**). Group I contains 25 members, Group II has 25, Group III 10, Group IV 11, Group V 16, and Group VI 13. All PcHDZIVs proteins are most closely related to those from *Salvia miltiorrhiza*, likely due to both being part of the true dicot family Lamiaceae, with a relatively recent divergence time. However, apart from sharing close phylogenetic relationships with *Salvia miltiorrhiza*, some proteins also show high kinship with genes related to GT development in other dicot plants. For instance, PcHDZIV1, 2, 20, 21 are closely related to AaHD8 from *Artemisia annua*; PcHDZIV4, 5, 6, 23, 25 are closely related to AaHD1 from *Artemisia annua*; PcHDZIV11, 12, 30, 31 are closely related to SlCD2 from tomato. These results indicate a potential close association between these genes and the development of GTs. Overall, the PcHDZIVs proteins show closer phylogenetic relationships with dicotyledonous plants than with monocotyledonous plants. However, PcHDZIV13, 14, 32, and 33 are highly homologous to OsRoc7 in rice, which may be a result of an ancient gene duplication event that predates the split between monocots and dicots.

### 3.5 Synteny analysis of patchouli *HD-ZIP IV* genes

**Figure 4:**
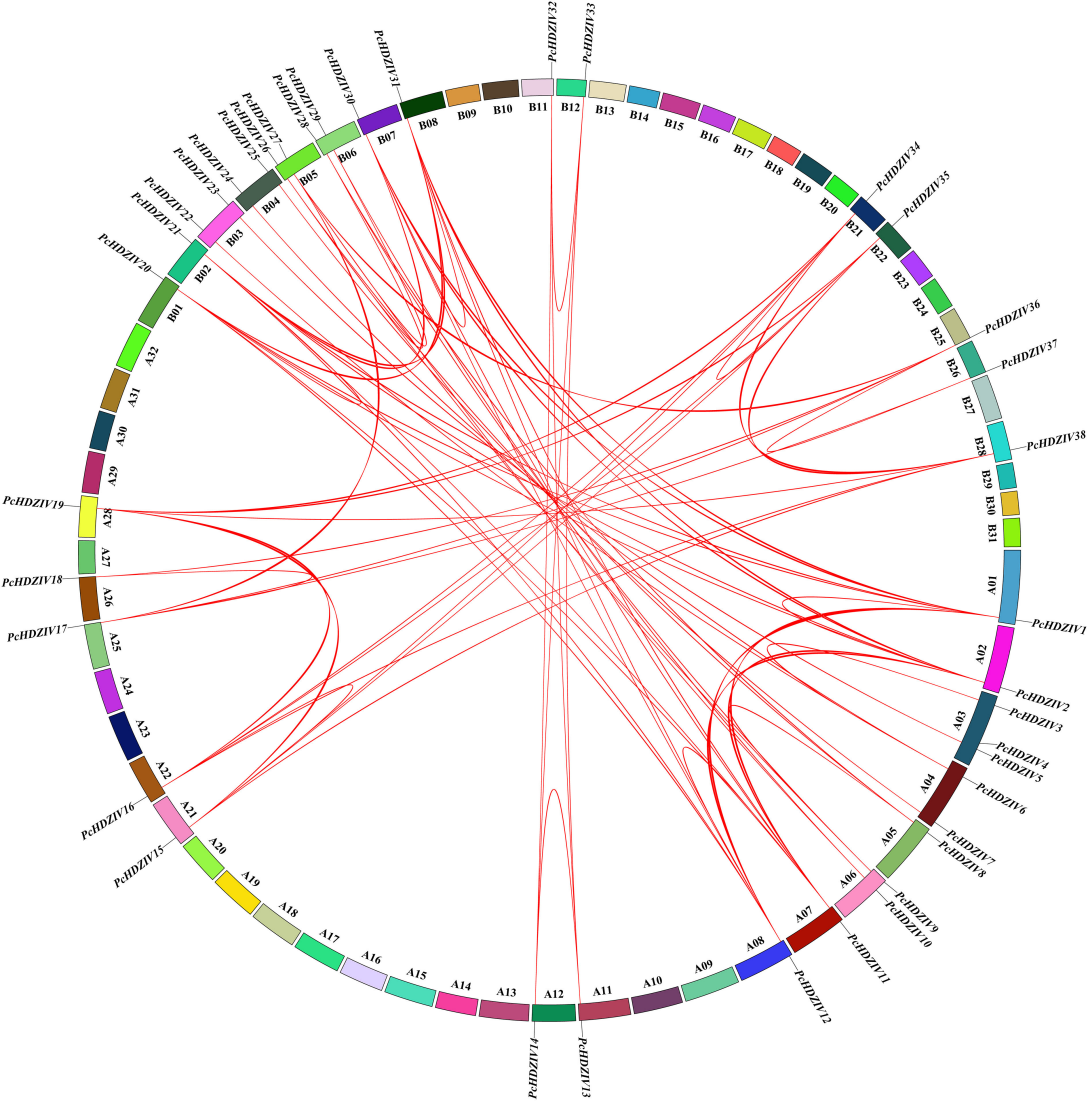
Chromosomal localization and synteny analysis of *PcHD-ZIP IV* genes. The inner circle displays the subgenome chromosome IDs in black font, and red lines indicate syntenic regions between genes.

The *PcHDZIVs* are symmetrically distributed across the A and B subgenomes of patchouli, located on chromosomes 1, 2, 3, 4, 5, 6, 7, 8, 11, 12, 21, 22, 25, 26, and 28 of the A subgenome and corresponding chromosomes of the B subgenome (**Figure 4**). The analysis of collinearity across *PcHDZIVs* revealed 69 gene pairs. Given their presence on distinct chromosomes, we believe that segmental duplication is the primary mechanism behind the evolution of *PcHDZIVs* gene family (**Figure 4**). Segmental duplication often results in family members being clustered or dispersed across the genome, providing redundant genes that enhance species adaptability. Evolutionary constraints on the *PcHDZIV* gene family can be deduced by analyzing the *Ka/Ks* ratio, which compares nonsynonymous and synonymous substitution rates. Only one gene pair, *PcHDZIV6/PcHDZIV25*, exhibits a *Ka/Ks* ratio exceeding 1, suggesting positive selection, possibly due to long-term genetic drift and mutation accumulation following duplication events. The *Ka/Ks* ratios of the remaining 68 gene pairs are all below 1 (**Supplementary Table 2**), which indicates that most of these genes have experienced purifying selection.

**Figure 5:**
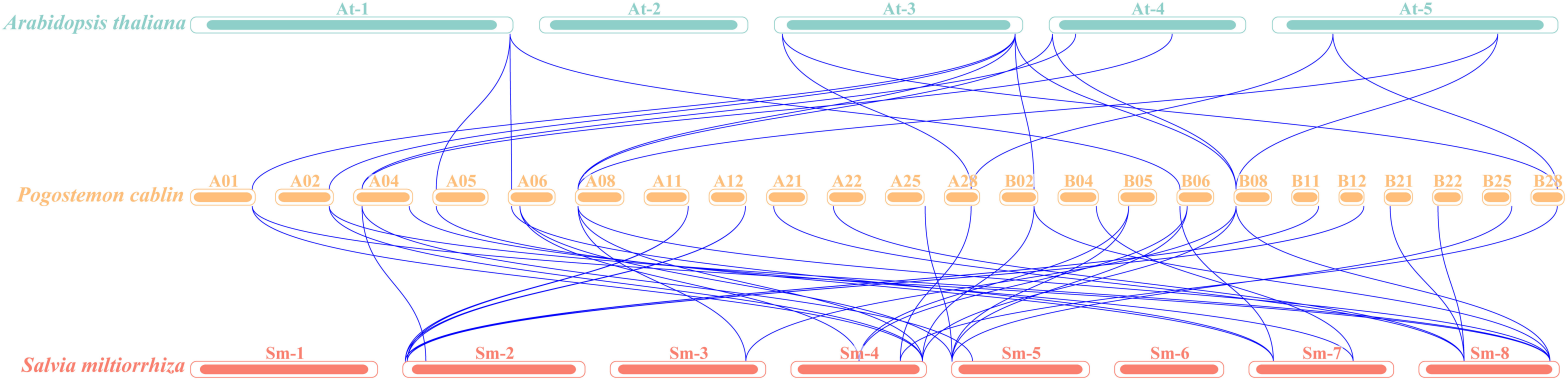
Synteny analysis of *HD-ZIP IV* genes among three genomes: *Arabidopsis thaliana* (Green), *Pogostemon cablin* (Orange), and *Salvia miltiorrhiza* (Red).

To explore the evolutionary origins and phylogenetic relationships of *HD-ZIP IV* genes across different species, a collinearity analysis was conducted among patchouli, *Arabidopsis*, and *Salvia miltiorrhiza*. There are 18 pairs of homologous *HD-ZIP IV* genes between patchouli and *Arabidopsis*, indicating that these 18 *HD-ZIP IV* genes in patchouli and *Arabidopsis* evolved from a common ancestor during the speciation process, while there are 41 pairs with *Salvia miltiorrhiza* (**Figure 5**). This suggests a closer phylogenetic relationship between patchouli and *Salvia miltiorrhiza HD-ZIP IV* genes, implying potential functional similarities.

### 3.6 Expression patterns of *HD-ZIP IV* genes in different tissues of patchouli

**Figure 6:**
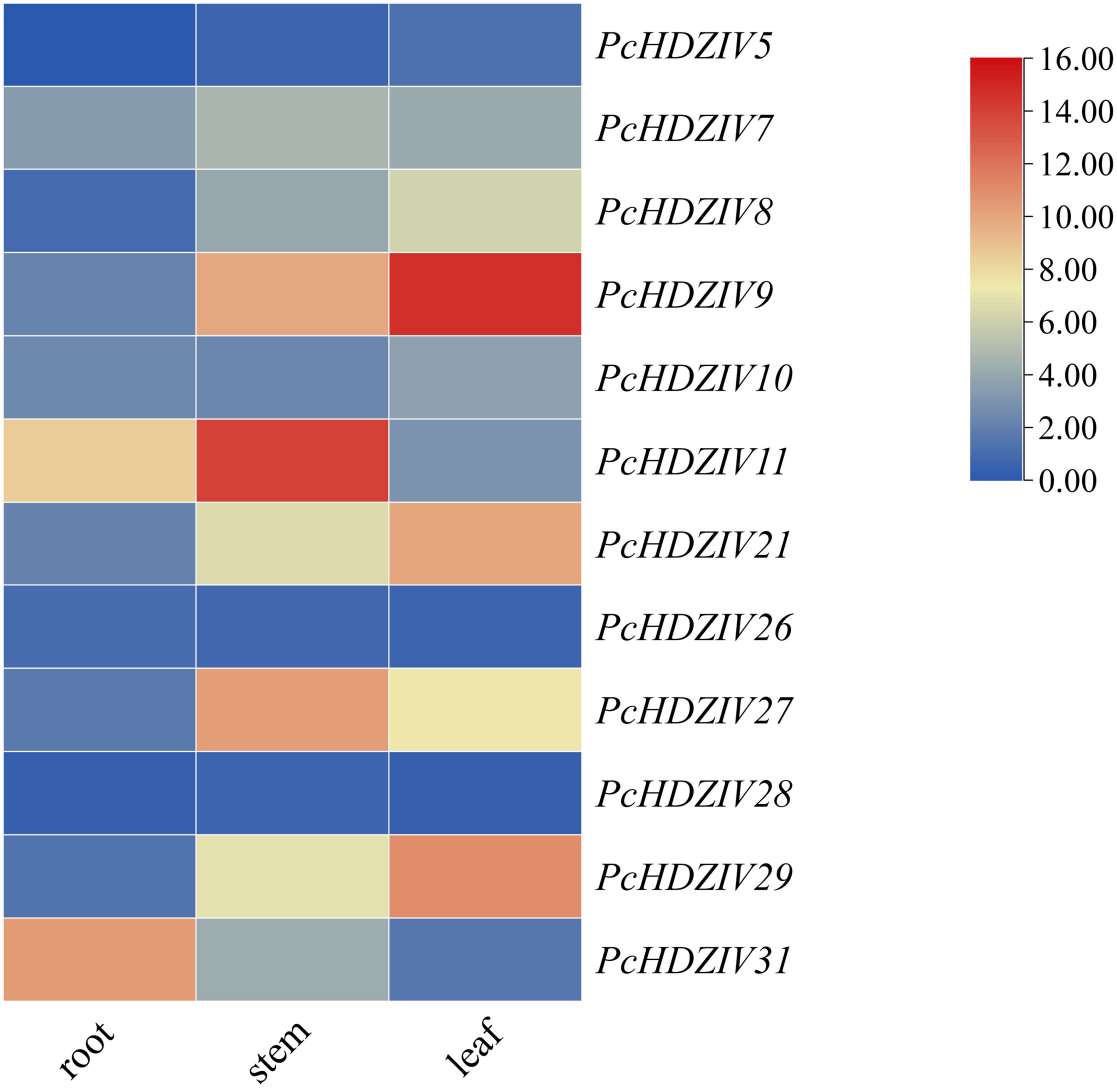
Expression pattern of the *HD-ZIP IV* gene in roots, stems, and leaves of Patchouli. Red indicates high expression levels, while blue represents low expression levels.

In order to analyze *PcHDZIVs* gene transcription levels in distinct patchouli tissues, we utilized RNA-seq datasets (BioProject ID: PRJNA606892, PRJNA607886) from roots, stems, and leaves to evaluate their expression. Results showed that *PcHDZIVs* exhibit pronounced tissue-specific expression, with their expression being significantly higher in leaves compared to other tissues (Figure 6). For instance, higher expression levels of *PcHDZIV5*, *8*, *9*, *10*, *21*, and *29* were observed in leaves relative to stems and roots, implying that these genes may be key players in regulating leaf development and function. Conversely, *PcHDZIV7*, *11*, *27*, and *28* were most highly expressed in stems, indicating a potential role in regulating specific stem functions. Additionally, *PcHDZIV26* and *31* were more highly expressed in roots. Distinct tissue-specific expression patterns were observed among the members of PcHDZIVs. Particularly, high expression levels in leaves were observed for most genes, aligning with the expression of *ATML* (Takada et al., 2013) and *PDF2* (Lu et al., 1996) in *Arabidopsis*, but differing from the reproductive organ-specific expression seen in cucumber (Fu et al., 2013) and immature reproductive organs in maize (Javelle et al., 2011). Overall, the expression patterns of the PcHDZIVs are highly correlated with tissue functions, suggesting their varied regulatory roles throughout the maturation and differentiation of different organs in patchouli. The findings establish a basis for further investigation into the roles of *PcHDZIV*s in driving tissue differentiation and functional specialization in patchouli.

### 3.7 Leaf-specific expression of HD-ZIP IV genes and their correlation with trichome density in patchouli

**Figure 7:**
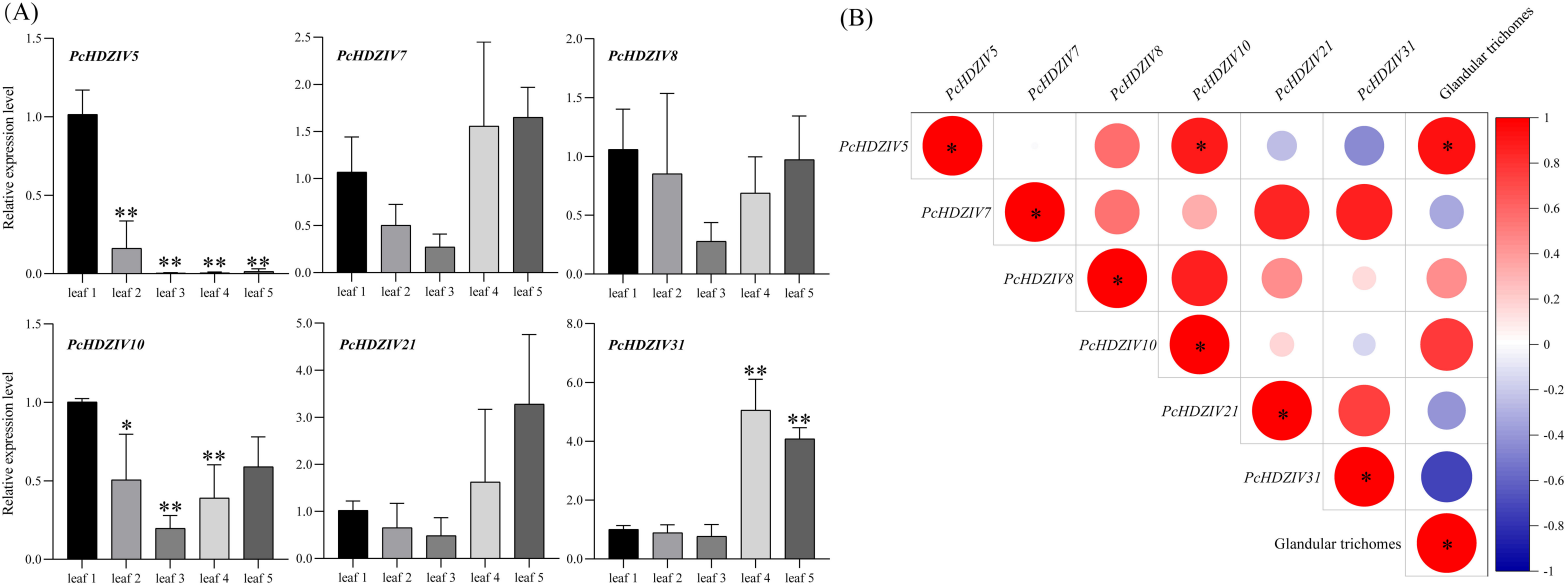
Relative expression analysis of *HD-ZIP IV* genes in different Leaf Orders of Patchouli and correlation heatmap with the number of GTs. **(A)** Asterisks above the bars indicate significant differences compared to the first leaf pair (*p < 0.05, **p < 0.01). **(B)** Asterisks denote significant correlations (*p<0.05). Relative expression analysis of *HD-ZIP IV* genes in different Leaf Orders of Patchouli and correlation heatmap with the number of GTs.

Based on transcriptional data from different tissues of patchouli, it is clear that leaves exhibit the highest expression levels for the majority of *PcHDZIV*s. Therefore, the expression of *PcHDZIV*s in the first to fifth pairs of leaves can be further analyzed using qRT-PCR. Transcriptome analysis led to the selection of four genes with high expression in leaves—PcHDZIV5, 8, 10, and 21—along with one gene each highly expressed in stems and roots, PcHDZIV7 and PcHDZIV31, to serve as target genes for studying the expression variations of *PcHDZIV*s in subsequent experiments. The findings revealed that *PcHDZIV5*, *8*, and *10* exhibited their peak expression levels in the first pair of leaves, *PcHDZIV31* reached its highest expression in the fourth pair, while *PcHDZIV7* and *21* showed maximal expression in the fifth pair of leaves. It was also observed that all genes had the lowest expression in the third pair of leaves (**Figure 7A**).

Research has shown that *HD-ZIP IV* genes contribute to the promotion of GT development in numerous plant species, including *Arabidopsis thaliana* (Nakamura et al., 2006), *Nicotiana tabacum* (Zhang et al., 2019), *Solanum lycopersicum* (Hua et al., 2021), and *Artemisia annua* (Yan et al., 2018). Therefore, to explore how *PcHDZIVs* relate to GT development, a heat map was created to show the Pearson correlation coefficients between the relative expression of *PcHDZIV*s in each leaf pair and the number of GTs. The findings demonstrated that *PcHDZIV5* exhibits positive correlation with both GT density and *PcHDZIV10* expression (**Figure 7B**). This suggests that *PcHDZIV5* may regulate GT development and interact with *PcHDZIV10*.

### 3.8 Virus-induced gene silencing of PcHDZIV5 and its effect on GTs

**Figure 8:**
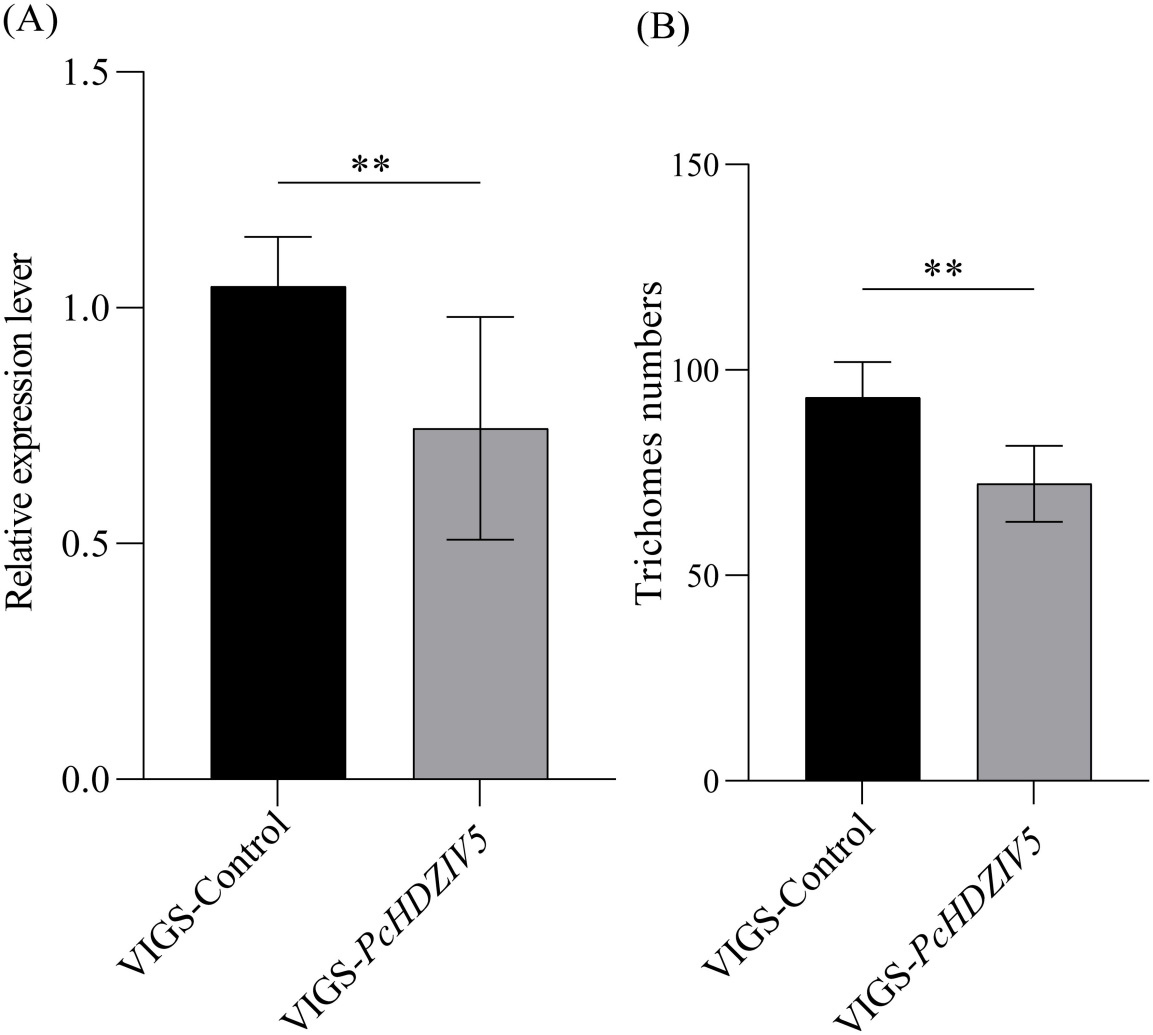
Analysis of virus-induced *PcHDZIV5* Silencing. **(A)** Relative mRNA expression levels of *PcHDZIV5* in *VIGS-PcHDZIV5* plants analyzed by qRT-PCR. **(B)** Effects of transient *PcHDZIV5* silencing on GTs in Patchouli. Asterisks above the bars indicate significant differences compared to the control group (**p<0.01).

To elucidate the role of the *PcHDZIV5* gene in the development of GTs in Patchouli, we conducted a virus-induced gene silencing (VIGS) experiment. After 14 days of silencing, leaf samples were collected to assess the expression of the target gene and the number of GTs. Through qRT-PCR analysis, we found that the expression of *PcHDZIV5* was significantly reduced (**Figure 8A**). At the same time, examination of the GTs on the abaxial side of the leaves revealed a significant decrease in GT density compared to the negative control group (pTRV2), with a reduction of approximately 23% (**Figure 8B**). These results further demonstrate that *PcHDZIV5* plays a crucial role in the growth and development of GTs in Patchouli.

### 3.9 Relative expression of patchouli HD-ZIP IV genes after plant hormone treatments

**Figure 9:**
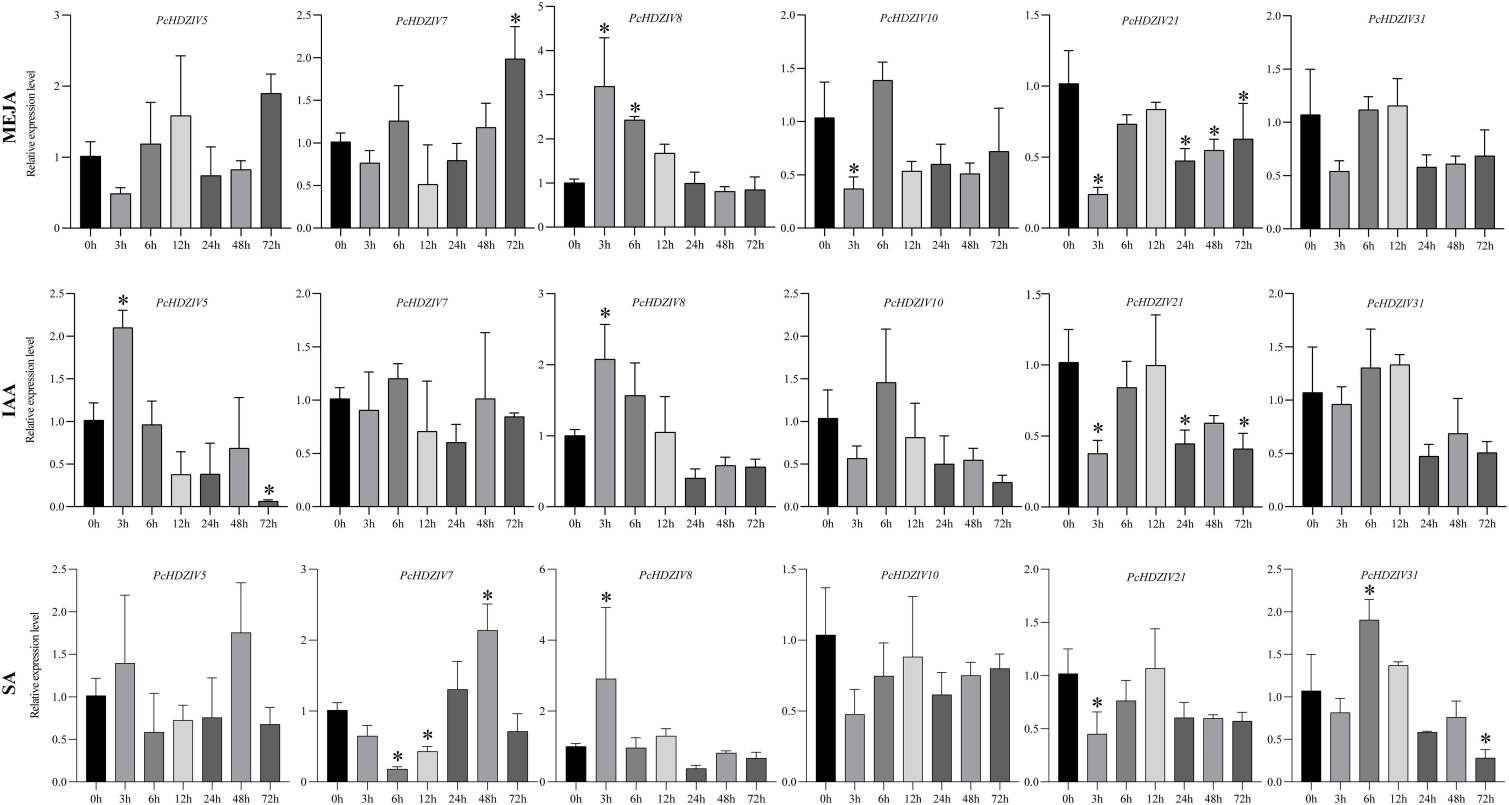
Relative expression analysis of the HD-ZIP IV gene under three hormone treatments. The relative expression levels of the HD-ZIP IV gene were measured at different time points (0 h, 3 h, 6 h, 12 h, 24 h, 48 h, 72 h) following treatment with methyl jasmonate (MeJA), indole-3-acetic acid (IAA), and salicylic acid (SA). All data were obtained from three biological replicates, and the results are presented as bar graphs with error bars representing standard deviation (SD). Asterisks at the top of the bars indicate significant differences compared to 0 h (*p < 0.05). The relative gene expression levels were calculated using the 2^−ΔΔΔCT^ method

The promoters of *PcHDZIVs*, according to predictive analysis, contain numerous cis-acting elements linked to exogenous hormones like MeJA, IAA, and SA. Consequently, the temporal expression patterns of six *PcHDZIV* genes in leaves subjected to hormone treatment were analyzed using qRT-PCR (**Figure 9**). The results show significant expression differences and temporal specificity among different *PcHDZIV* genes under the three hormone treatments. For example, *PcHDZIV5* was upregulated at 12 hours and 72 hours after MeJA treatment and showed significant upregulation at 3 hours after IAA treatment, peaking at 48 hours post-SA treatment. *PcHDZIV7* showed significant upregulation in the later stages (48h to 72h) of MEJA and SA treatments, indicating a delayed response pattern, suggesting its role in the later stages of hormone signal regulation. *PcHDZIV*8 was significantly upregulated at 3 hours during MeJA and SA treatments and maintained high expression in the early (3h) and mid-stages (6h) of IAA treatment, indicating its sensitivity to multiple hormones. Additionally, some genes were significantly downregulated early after hormone treatment, such as *PcHDZIV21*, which showed a significant decrease at 3 hours during all three hormone treatments, but some genes, like *PcHDZIV31*, recovered and increased in the mid-stages. While some genes showed consistent expression patterns across the three hormone treatments, such as *PcHDZIV8*, which exhibited an “initial rise then decline” trend, *PcHDZIV10*, *PcHDZIV21*, and *PcHDZIV31* displayed a “decline then rise then decline” dynamic pattern. However, *PcHDZIV5* and *PcHDZIV7* had different response trends under various hormone treatments, indicating they may have specific responses to different hormonal signals.

## 4 Discussion

Through a comprehensive analysis of *Pogostemon cablin* genome database, identifying 38 non-redundant *PcHDZIV* genes and comprehensively investigating their characteristics. The gene structure and conserved motifs of them are generally similar, reflecting functional similarity among the family members. However, the absence of specific motifs, such as motif 3 in *PcHDZIV27* and motifs 1 and 5 in *PcHDZIV34*, combined with differences in exon numbers across genes, indicates potential subfunctionalization or adaptations to distinct environmental conditions.

In the phylogenetic branches, the patchouli *HD-ZIP IV* gene family includes a greater quantity of paralogous genes. In contrast to other dicot plants, patchouli exhibits a greater level of gene duplication in these branches, with an increased number of gene copies. This can be attributed to a whole genome duplication (WGD) event that patchouli experienced about 3.3 million years ago and a burst in long terminal repeat retrotransposon (LTR-RT) amplification around 1.1 million years ago. These events led to chromosomal doubling and significant gene amplification. Additionally, unlike other polyploid hybrid plants that primarily reproduce sexually, patchouli, which relies mainly on asexual reproduction, shows no significant gene loss between subgenomes A and B (Doyle et al., 2008; Yoo et al., 2014; Shen et al., 2022). Phylogenetic analysis of these genes aids in revealing evolutionary relationships and provides a basis for predicting functions in specific plant species. Most PcHDZIVs proteins and other dicots cluster together, indicating that these genes likely have a conserved regulatory role across dicotyledons. *HD-ZIP IV* genes are related to GT development across multiple species. In *Artemisia annua*, *AaHD8* interacts with *AaMIXTA1* to form an HD-ZIP IV/MIXTA complex, subsequently regulating the activity of *AaHD1*. Through the regulation of *AaGSW2* expression, *AaHD1* promotes the onset of GTs, advancing the formation and development of *Artemisia annua* GTs (Yan et al., 2017, 2018). The *SlCD2* gene in *Solanum lycopersicum* plays a role in GT development. A loss of function in this gene causes the sticky peel (pe) phenotype, characterized by a reduction in type VI GTs to one-third of the wild-type level (Nadakuduti et al., 2012). Therefore, genes such as *PcHDZIV1*, *2*, *4*, *5*, *6*, *20*, *21*, *23*, *25*, which are closely related to *AaHD1* and *AaHD8* from *Artemisia annua*, and P*cHDZIV11*, *12*, *30*, *31*, which are closely related to the tomato *SlCD2*, may also participate in regulating GT development. The findings serve as valuable clues for advancing subsequent research on gene function.

In bananas, HD-ZIP IV gene activity exhibits a linear upregulation or downregulation trend from juvenile leaves to mature leaves (Pandey et al., 2016). In contrast to the pattern observed in bananas, where *PcHDZIVs* expression changes linearly during leaf maturation, patchouli displays a unique trend, first declining and then rising. Among younger leaves (e.g., leaf 1 through leaf 3), *PcHDZIVs* expression gradually diminishes, hitting its lowest level in leaf 3. This phenomenon aligns with the higher activity of physiological processes such as epidermal hair development in juvenile tissues in patchouli, suggesting that they are closely involved in epidermal hair development and its metabolic processes. Compared to the expression patterns observed in younger leaves, *PcHDZIVs* display varying trends in leaf 4 and leaf 5, which implies their involvement in multiple regulatory roles during various developmental stages of patchouli. Furthermore, the association between gene expression profiles and GT density demonstrates a significant positive link between the expression of *PcHDZIV5* and GT density, indicating its potential role in modulating GT formation. According to the phylogenetic relationship, *PcHDZIV5* is closely related to *AtHDG5*, and like *AtHDG5*, it may regulate its expression in the L1 layer through the GL1/GL3 regulatory module and cooperate with additional HD-ZIP IV genes, like *PcHDZIV10*, to jointly regulate GT development (Nakamura et al., 2006). Since GTs primarily store secondary metabolites, such as pogostone, which are directly tied to the economic significance of plants (Turner et al., 2000; MaChado et al., 2006), these results have important potential applications.

The CRE analysis of the *PcHDZIVs* promoters indicates significant regulatory potential in light response, hormone regulation, and stress response. Notably, all gene promoters contain light-responsive elements (such as Box 4), suggesting that they might be crucial in regulating plant photomorphogenesis. In addition, the broad presence of ABA-responsive ABRE elements and stress-related motifs (e.g., TC-rich repeats) underscores the significant role of these genes in abiotic stress responses. Plant hormones are key regulators of resistance to a variety of stress conditions, including both living organism-induced and environmental stressors, during growth and development (Gómez-Cadenas et al., 2014). Induction of *PcHDZIVs* expression by exogenous hormones (MeJA, SA, IAA) underscores their dynamic responsiveness to internal plant signaling molecules. For example, *PcHDZIV7* is significantly upregulated in the early stage (3 hours) under MeJA, IAA, and SA treatments, suggesting its involvement in the early signaling pathways of these hormones. Moreover, most genes in this study exhibited consistent expression patterns under the three hormonal treatments, showing either a rise followed by a fall or an initial dip, a subsequent increase, and a final decline. Overall, these genes demonstrate a remarkable temporal-specific expression pattern in response to exogenous hormone treatments. The response characteristics of different hormone types further reveal the functional diversity of these genes in hormone signaling. IAA treatment tends to regulate earlier stages, while MeJA and SA may mediate longer-term dynamic regulation. These results illuminate the participation of *PcHDZIVs* in hormone signaling mechanisms and lay a foundation for further exploration of their related regulatory networks.

In *Artemisia annua*, the formation of GTs is mainly governed by jasmonic acid (JA). *AaJAZ8*, a repressor in the JA signaling cascade, suppresses the activity of *AaHD1*, leading to a reduction in GT density (Yan et al., 2017). In the phylogenetic tree, it can be observed that *AaHD1* is closely related to *PcHDZIV5*. Correlation analysis between gene expression levels and GT density, along with virus-induced gene silencing (VIGS) assays, further confirmed the critical role of *PcHDZIV5* in GT development. Additionally, the significant upregulation of *PcHDZIV5* expression following MeJA treatment suggests that hormone signaling in patchouli may regulate *PcHDZIV5* transcription, thereby indirectly promoting GT development. Based on the analysis of CREs, it can be further inferred that hormone signals may precisely regulate *PcHDZIV5* through specific CREs in the promoter (such as the TCA-element for salicylic acid, the CGTCA-motif for jasmonic acid, and the TGA-element for auxin). This regulation may activate downstream signaling networks, promoting GT differentiation and functional development. As an important storage site for patchouli’s secondary metabolites, the formation of GTs is orchestrated by the interplay of multiple hormonal signals. This discovery highlights the dual significance of *PcHDZIV5* in both GT development and stress adaptation in patchouli, establishing a theoretical foundation for subsequent investigations on its molecular mechanisms.

## 5 Conclusion

This study carried out a thorough genome-wide analysis of the HD-ZIP IV gene family in *Pogostemon cablin* and identified 38 *PcHDZIV* genes. These genes were categorized into six subgroups based on phylogenetic analysis. Further exploration of the gene structure, composition of conserved motifs, cis-regulatory elements, and synteny relationships disclosed the evolutionary traits and potential functions of this gene family. Expression pattern and correlation analyses revealed that certain *PcHDZIV* genes are highly expressed in specific tissues, particularly in leaves. Notably, *PcHDZIV5* expression exhibited a significant positive correlation with GT density. Moreover, virus-induced gene silencing (VIGS) of *PcHDZIV5* resulted in a reduction in its transcript abundance and a significant decrease in the number of GTs in patchouli. These findings further confirm that *PcHDZIV5* plays a key regulatory role in GT development. Additionally, most *PcHDZIV* genes exhibited significant dynamic responses to exogenous hormone treatments (such as MeJA, IAA, and SA). For instance, *PcHDZIV5* expression was upregulated during the 3 to 12-hour period following *MeJA* and *IAA* treatments, while it peaked at 48 hours following *SA* treatment. Based on expression patterns and the distribution of CREs, it is hypothesized that hormone signals may regulate GT development through the mediation of the *PcHDZIV5* gene. The findings of this research lay a foundation for further investigations on the *PcHDZIVs*, and highlight their potential functions in the development of GTs and the morphogenesis of leaves in patchouli.

## Data Availability

The datasets presented in this study can be found in online repositories. The names of the repository/repositories and accession number(s) can be found in the article/**Supplementary Material**.
